# Simulation and Optimization of a Planar-Type Micro-Hotplate with Si_3_N_4_-SiO_2_ Transverse Composite Dielectric Layer and Annular Heater

**DOI:** 10.3390/mi13040601

**Published:** 2022-04-12

**Authors:** Guangfen Wei, Pengfei Wang, Meihua Li, Zhonghai Lin, Changxin Nai

**Affiliations:** 1 School of Information and Electronic Engineering, Shandong Technology and Business University, Yantai 264005, China; limeihua@sdtbu.edu.cn (M.L.); zhlin@sdtbu.edu.cn (Z.L.); 2 Solid-State Institute, Chinese Research Academy of Environmental Sciences, Beijing 100012, China; naicx@126.com

**Keywords:** micro-hotplate, thermal simulation, temperature distribution, mechanical deformation

## Abstract

Micro-hotplates (MHPs) have become widely used basic structures in many micro sensors and actuators. Based on the analysis of the general heat transfer model, we propose a new MHP design based on a transversal composite dielectric layer, consisting of different heat transfer materials. Two general proven materials with different thermal conductivity, Si_3_N_4_ and SiO_2_, are chosen to form the composite dielectric layer. An annular heater is designed with a plurality of concentric rings connected with each other. The relationship between MHP performance and its geometrical parameters, including temperature distribution and uniformity, thermal deformation, and power dissipation, has been fully investigated using COMSOL simulation. The results demonstrate that the new planar MHP of 2 μm thick with a Si_3_N_4_-SiO_2_ composite dielectric layer and annular heater can reach 300 °C at a power of 35.2 mW with a mechanical deformation of 0.132 μm, at a large heating area of about 0.5 mm^2^. The introduction of the composite dielectric layer effectively reduces the lateral heat conduction loss and alleviates the mechanical deformation of the planar MHP compared with a single SiO_2_ dielectric layer or Si_3_N_4_ dielectric layer.

## 1. Introduction

Micro-hotplates (MHPs) fabricated by micromachining processes have proven to have the advantages of miniaturization, low power consumption, fast thermal response, and easy integration with other devices [1], which have become a widely used basic structure in micro gas sensors, micro gas flowmeters, infrared light emitters and sensors, and micro thermometers [2,3,4]. In these devices, MHPs are performed to provide mechanical supports with a suitable operating temperature for functional materials or structures [5]. Research on MHPs dates back to the 1990s and deals with the simulation, manufacturing processes, heater shape and materials of heaters, structures and materials of plates, and heat transfer theory. The aim is to pursue miniaturization, low power consumption, uniform temperature distribution, good mechanical stability, and easy manufacturing of MHPs [6,7,8,9].

Fabrication technology is the most critical to batch production, which has contributed to the tremendous development of surface micromachining and bulk micromachining technologies [10,11,12,13,14,15]. Surface micromachining has the advantages of low fabrication cost and easy integration with CMOS (Complementary Metal Oxide Semiconductor) circuits, thus giving birth to many microbridge MHPs, cantilever MHPs, and microbridge-supported MHPs. However, with the development of bulk micromachining, the above structures can be easily fabricated by silicon etching processes, which have been standard micromachining processes. Today, many new fabrication methods have been proposed to make MHPs more reliable and improved. A Polymorphs technology can eliminate the need for alignment masks during post-processing, making the fabrication of MHPs easier, even though the design rules are intentionally violated [16]. A controlled two-step gate notch-etching method improves the performance of MHPs in gas sensors [17]. Simultaneously, a modified front-end etching process was introduced to fabricate a bridge-type MHP with a compact active region of 4 μm × 8 μm powered less than 10 mW [18].

Based on the rapidly developing micromachining technology, various geometries of MHPs have been investigated in literature, as shown in Table 1. It can be observed that planar-type (also called membrane type) MHPs can provide larger active heating areas than suspended type (also called microbridge type) MHPs. Here, planar MHPs usually have their active heating areas completely attached to the base, while for suspended MHPs, the active heating areas are supported by 1 to n bridges [19,20,21,22], such as cantilever (one anchorage point), bridge type (two anchorage points), and plate hanging by several bridges (more than two). The most typical suspended MHP is the four-bridge supported plate, showing a good symmetry and very low power for heating. However, as they are anchored by several points, the suspended-types are easily damaged by the post fabrication process, which reduces the yield and reliability of devices. Accordingly, the planar-type structure shows good supportive performance, but its heating power is significantly high due to the absence of any thermal isolation window. More support bridges or thermal isolation windows were used to improve the mechanical properties of the suspended structure, but the disadvantage is the increased fabrication difficulty [23,24]. Silicon island structures have also been used for planar MHPs to obtain better temperature uniformity and better mechanical properties, but heat losses are increased [25]. Hence, new planar MHP designs with high heating efficiency and good mechanical support are critical.

Pattern shape and material of the heater have been issues of MHP. The shape of the heaters has been investigated, affecting the temperature distribution of MHP. An optimized serpentine heater with up to 86.64% coverage on a rectangular film has been reported [30]. Two semicircular heaters have also been designed on a circular plate to achieve a temperature of 300 °C at 8 mW [33]. Heater materials, such as tin, polysilicon (poly-Si), molybdenum, platinum (Pt), tungsten (W), and some alloys, have been explored [28,29,31,32,34,35,36]. Ideally, the heater material should have high thermal conductivity, low thermal expansion coefficient, high melting point, and compatibility with standard silicon manufacturing processes. Polysilicon has been the most widely used material for heaters due to its compatibility with CMOS processes. However, the resistivity of heaters is unstable due to the instability of polysilicon grains at high temperatures. Platinum has proven to be a good candidate for MHP heaters because of its satisfied performances, and has been widely adopted, as shown in Table 1.

Dielectric layers for heaters are often fabricated as interlayers, which are actually the main part of the MHP. SiO_2_ and Si_3_N_4_ are often chosen as the dielectric materials because of their well-established processes in most MHPs [29]. In recent years, new materials such as SiC, polyamide and porous silicon have also been proposed for MHPs as insulation layers [27,37,38], but the fabrication processes of these materials are too complex and still in research. Because SiO_2_ and Si_3_N_4_ have internal and tensile stresses, respectively, they can mitigate the thermal deformation of MHPs if used in combination [39,40]. Therefore, most MHPs use vertical-layered SiO_2_/Si_3_N_4_ as plate materials, as shown in Table 1.

Simulation has proven to be a useful technique to study, not only electrical, but also heat transfer and mechanical properties, with the help of finite element analysis software, such as ANSYS, COMSOL, and ConventorWare [41,42]. An electrical heating model is generally adopted to simulate the operation of MHPs [26]. Based on the designed model, the effect of the thickness of the Si_3_N_4_ layer on the temperature, mechanical stability, and power consumption of the MHP can be predicted by electro-thermo-mechanical multiphysics field simulations [43]. An array of MHPs can be analyzed and the dimensions of MHPs can also be optimized [44]. Various heater geometries were simulated to determine which geometry would provide a more uniform temperature distribution [45]. Parameters of MHPs were optimized by simulation, such as the thickness of the MHP substrate [46] and size of the insulation nitride area of the MHP [47]. Several simulated MHP designs have been listed in Table 1 for comparison, which further proves simulation to be a very effective way to optimize and verify MHP designs.

Inspired by the above literature review, it can be observed that MHPs with high heating efficiency and good mechanical support are still hot research points. Planar-type MHPs demonstrate better mechanical support and stability, while have more power consumption than the suspended-type. To resolve this dilemma, new heat isolation approach and structure design are demanded. This paper proposes a new planar MHP design introducing a transverse composite dielectric layer with an annular platinum heater based on the most common and fundamental micromachining process. A systematic simulation of the design is performed with the COMSOL software. Section 2 presents the thermal analysis model and design methodology. Section 3 shows the validation of the model and the detailed results of the parameter simulation. Finally, Section 4 concludes the paper.

## 2. Analytical Models and Design Methods

### 2.1. Heat Transfer Model of MHP

**Figure 1:**
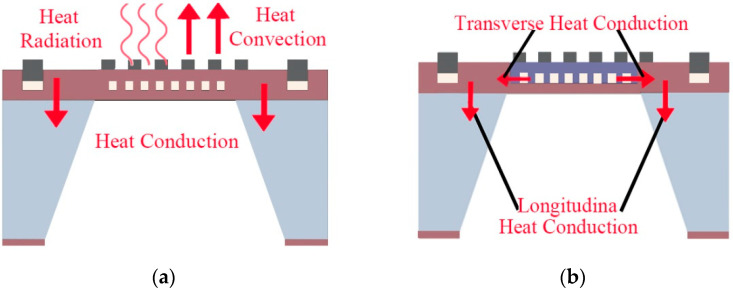
(**a**) Heat transfer model of MHPs. (**b**) Heat conduction model of MHPs.

Currently, most researchers believe that the heat transfer paths of MHP can be divided into three main categories: conduction, radiation, and convection, as shown in Figure 1a [48]. Assuming that the total heat of the MHP is defined as $Q_{tot}$, the ambient temperature is $T_{amb}$, and the operating temperature of the MHP is $T_{hot}$, according to Fourier’s heat transfer law and Stefan-Boltzmann’s law, the simplified MHP heat transfer equation, including thermal radiation and convection, is defined as: [49,50]:

(1) $$Q_{tot}=G_{m}\lambda_{m}\left(T_{hot}-T_{amb}\right)+(G_{air}\lambda_{air}+G_{airf}h_{f})\left(T_{hot}-T_{amb}\right)+G_{rad}K_{B}\epsilon_{T}\left(T_{hot}^{4}-T_{amb}^{4}\right)$$

where $\lambda_{m}$ and $\lambda_{air}$ are the thermal conductivity of the membrane material and air, respectively. $h_{f}$ is the air convection coefficient, $\epsilon_{T}$ is the radiation coefficient, and $K_{B}$ is the Boltzmann coefficient. $G_{m}$ is the geometric factors of heat conduction related to the membrane structure. For a multi-layer composite membrane of radius $r_{m}$ embedded with a heating zone of radius $r_{h}$,

(2) $$G_{m}=\frac{2\pi d}{\ln\left(\frac{r_{m}}{r_{h}}\right)}$$

where d represents the thickness of the membrane [49]. In addition, the thermal conductivity of a membrane composed with *n* layers is defined as

(3) $$\lambda_{m}=\frac{\sum_{i=1}^{n}\lambda_{i}\times d_{i}}{\sum_{i=1}^{n}d_{i}}$$

where $d_{i}$ and $\lambda_{i}$ are the thickness and the thermal conductivity of the *i*th layer, respectively. Generally, $G_{air}=4\pi r_{h}$, $G_{airf}=\pi r_{h}^{2}$, and $G_{rad}$ are geometric factors related to the membrane structure corresponding to the thermal conduction of air, convection of air, and thermal radiation.

The air convection coefficient $h_{f}$ can be obtained by Equation (4), where $N_{u}$ represents the Nusselt number [51].

(4) $$h_{f}=\frac{N_{u}\lambda_{air}}{r_{h}}$$

For a free convection heat transfer, the Nusselt number is related to the thermal conductivity and heat capacity of the air, the air density, temperature, and the geometric factors of the MHP, which is still a quite nonlinear and controversial term. Most studies of the MHPs empirically set this value as a constant term [52].

Based on these models, heat conduction in composite media and heat transfer in microscale air gaps were analyzed and investigated [53,54]. It has been noted that the effect of heat loss due to radiation can be neglected; heat conduction should be the dominant form of heat transfer when the temperature is below 500 °C [55,56]. Outer air flow greatly influences the thermal convection, which mostly affects the air convection coefficient. For simplicity, natural air convection is usually considered in experiments [49,54].

### 2.2. Thermal Deformation Computation

Based on the heat transfer model, temperature distribution of the MHP can be acquired when inputting the heating power, $Q_{tot}$. However, due to the different thermal expansion of materials, the thermal stress caused by the temperature variation may lead to large thermal strain, according to the thermal stress and mechanical static equation, as shown in Equations (5) and (6) [57].

(5) $$\sigma =E\left(\varepsilon_{T}-\varepsilon_{0}\right)$$

(6) $$\varepsilon_{T}=\alpha \Delta T$$

where $\varepsilon_{T}$, $\varepsilon_{0}$, E, α, and ΔT are the strain at temperature *T*, initial thermal strain, Young’s modulus matrix, thermal expansion coefficient, and temperature difference, respectively.

This thermal strain is related with the displacement of material, which is usually expressed by the Cauchy equation. Its simplified form is shown as Equation (7) [58],

(7) $$\varepsilon_{all}=\frac{1}{2}\left[{\left(\nabla \vec{u}\right)}^{T}+\nabla \vec{u}\right]$$

where $\varepsilon_{all}$ represents the total strain (generally approximates to the $\varepsilon_{T}$ if only the thermal strain is considered), $\nabla \vec{u}$ represents the displacement vector, and *T* represents the transpose. This displacement is defined as the mechanical deformation, or thermal deformation, as it is majorly caused by the temperature variation.

The basic thermal transfer and thermal deformation models have been integrated into the multiphysics software of COMSOL. Hence, the temperature distribution, thermal deformation, and power consumption of the designed MHPs are simulated by COMSOL in the following sections.

### 2.3. Presumption Verification

More intuitively, heat conduction in the MHPs can be equated to the longitudinal interlayer conduction and transverse conduction, as shown in Figure 1b. Combining the heat transfer equation, it can be concluded that reducing the heat contact area or heat transfer coefficient of MHP can prevent the heat conduction loss.

To verify the presumption, three MHP models (Types A–C) were simulated with COMSOL at the specific power consumption of 35 mW, as shown in Table 2. Their active heating areas were uniformly set to 800 μm × 800 μm, consisting of a 1.4 μm thick SiO_2_ support layer and 0.6 μm thick Si_3_N_4_ dielectric layer. The same Pt heater was embedded in the Si_3_N_4_ dielectric layer. The other dimensions and parameters remained the same for all models, except with the addition of air windows.

Type A is a most common kind of planar MHP with two vertical layers of SiO_2_ and Si_3_N_4_, reaching 158 °C at 35 mW heating power, as shown in the first row of Table 2. However, if 2 and 4 etching windows are added to the plate, which is Type B and C, higher temperatures of 174 °C and 199 °C will be reached at the same heating power, respectively. Obviously, this demonstrates that the etched window greatly isolates the transverse heat conduction in the plate. These are actually the suspended type (microbridge) MHPs.

However, mechanical deformation also increases severely when the window is increased from Type A to Type C. Thermal deformation will cause a degradation of the functional material and definitely leads to a low stability and short lifetime of the device. This dilemma between temperature and mechanical deformation inspired the design of Type D in Table 2. By designing a transverse composite dielectric layer with different thermal conductivity, planar type MHPs can reach higher temperatures and lower thermal deformation with the same heating power. Novel materials with a low thermal conductivity can be introduced, but the fabrication process may be complexed. It is worth noting that SiO_2_ and Si_3_N_4_ are widely used in MHPs as the most common materials, but with different thermal conductivity.

### 2.4. Structural Design of the New MHP

**Figure 2:**
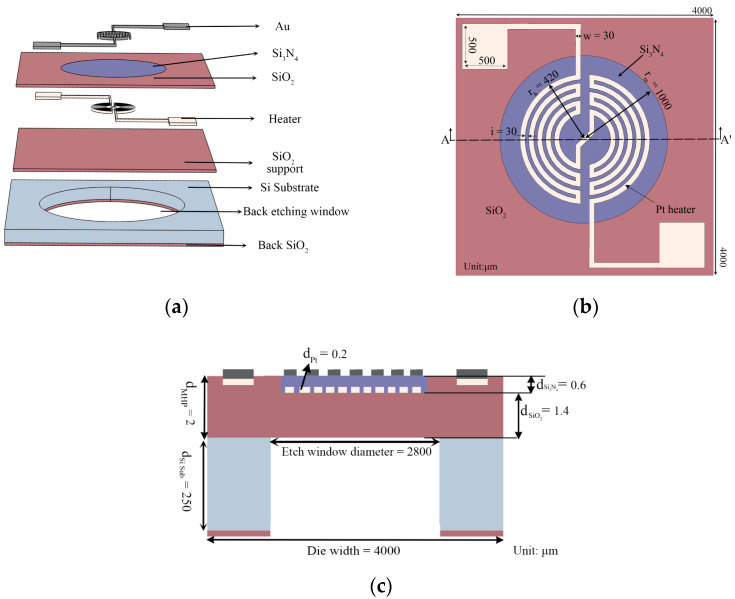
(**a**) The designed MHP. (**b**) The Si_3_N_4_- SiO_2_ composite dielectric layer with the annular heater. (**c**) Cross-sectional view of the MHP on the horizontal line A-A’ shown in (**b**).

The newly designed planar type MHP with composite dielectric layer is shown in Figure 2. The plate contains a SiO_2_ support layer, a composite dielectric layer of Si_3_N_4_ and SiO_2_, an annular heater, and a comb interdigital electrode. SiO_2_ and Si_3_N_4_ are two very basic and mature dielectric materials in silicon fabrication processes, but they exhibit very different thermal conductivity properties. Their thermal conductivity is 1.4 Wm^−1^K^−1^ and 20 Wm^−1^K^−1^, respectively, while their thermal expansion coefficients are 0.5 × 10^−6^ K^−1^ and 2.3 × 10^−6^ K^−1^, respectively. Since Si_3_N_4_ shows better thermal conductivity and expansion than SiO_2_, it is designed as a central heating zone for thermal conductance, while the SiO_2_ layer is designed around it for better thermal insulation. Hence, a composite dielectric layer with Si_3_N_4_ encircled by the SiO_2_ layer is obtained. The layers of MHP are shown in Figure 2a.

Figure 2b,c shows the planar view of the Si_3_N_4_-SiO_2_ composite dielectric layer with the annular heater and the cross-sectional view of the MHP on the transverse line A-A’. Initially, the chip size was set to 4000 μm × 4000 μm, the total plate thickness was set to 2 μm, including the SiO_2_ support layer (1.4 μm thick) and the Si_3_N_4_-SiO_2_ composite dielectric layer (0.6 μm thick). The radius $r_{m}$ of the Si_3_N_4_ zone of the dielectric layer was initially designed to be 1000 μm. The Pt heater was embedded in the dielectric layer with an initial radius $r_{h}$ of 420 μm, to balance the power consumption and heating area demands [59]. In addition, its width w and space i were initially set to 30 μm.

### 2.5. The Process Design

**Figure 3:**
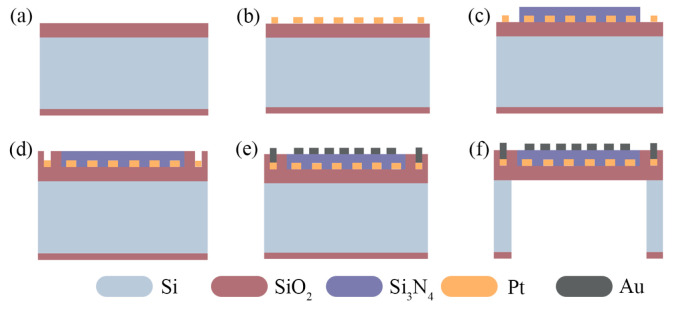
The simplified fabrication steps of the designed planar MHP. (**a**) Deposition of SiO_2_. (**b**) Sputter of Pt heater. (**c**) Deposition of central Si_3_N_4_. (**d**) Deposition of external SiO_2_. (**e**) Sputter Au electrode. (**f**) Backside etching.

The simplified MHP fabrication steps are designed as follows and shown in Figure 3.

(a) Thermal oxidation of the silicon substrate, and the deposition of low-stress 1.4 μm thick SiO_2_ as the support layer.

(b) Sputtering of 0.2 μm thick Pt, photo-lithography, and etching of Pt as the heater.

(c) Patterning the circular Si_3_N_4_ zone and deposition of the low-stress 0.6 μm thick Si_3_N_4_ layer.

(d) Patterning the outer SiO_2_ layer and deposition of low-stress 0.6 μm thick SiO_2_ to compose the dielectric layer with Si_3_N_4_ in step (c).

(e) Patterning, sputtering, and lift-off Au as the comb electrode.

(f) Patterning and back-side etching to release the planar MHP structure.

In order to enhance the adhesion of Pt, a layer of TiO_2_ with a thickness of 5–15 nm is usually sputtered before and after Pt deposition [33]. As there will be great stress at the Si_3_N_4_-SiO_2_ junction in the composite dielectric layer, high temperature annealing is required to relieve the stress after step (d). In addition, to remove the overlap at the interface of the composite film, Chemical Mechanical Polishing (CMP) is required for planarization. The total fabrication steps of the MHP are completely general and standard MEMS processes.

## 3. Simulation and Optimization

### 3.1. Validation of the MHP Model

As shown in Figure 2, the designed MHP is based on a silicon substrate with a thickness of 250 μm. The material parameters used are shown in Table 3, and the heat convection coefficient of air $h_{f}$ was set to 30 (W m^−2^·K^−1^) [49]. Unless stated separately, the input power of the solid heat transfer mode of COMSOL was set to constant in the following experiments.

For the MHP shown in Figure 2, the temperature distribution of the MHP at a power of 34 mW was obtained by the COMSOL software. The center temperature of the MHP was 319 °C, the edge temperature of the MHP was 24 °C, and the temperature difference ΔT is 295 °C. Based on the heater transfer models in Section 2, the theoretical solid heat conduction power, air heat conduction power, and air heat convection power consume 27.8 mW, 0.7 mW, and 4.9 mW, respectively. The total theoretical power consumption is 33.4 mW, which is close to the input power in COMSOL.

### 3.2. Dielectric Layer Analysis

**Figure 4:**
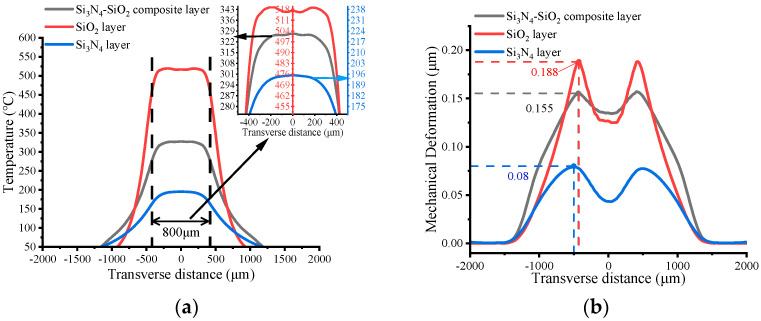
Comparison of MHP with different dielectric layers: (**a**) Temperature distribution with an enlarged view of the circular area. (**b**) Mechanical deformation due to thermal stresses. All the geometric parameters are the same; the thickness of MHPs is 2 μm.

The temperature distribution and thermal deformation of the Si_3_N_4_-SiO_2_ composite dielectric layer-based MHP were analyzed by establishing the physical field of solid heat transfer. Results are shown in Figure 4a,b. For comparison, the dielectric layers of pure SiO_2_ and pure Si_3_N_4_ were also explored. Excepting the material of the dielectric layer, all other setups are the same as in Figure 2. Heating power was set to constant 41 mW. It can be observed that the plate embedded with a pure SiO_2_ dielectric layer reaches 500 °C, but has a non-uniform temperature distribution in the high temperature region and a mechanical deformation of up to 0.188 μm. The plate embedded with a Si_3_N_4_ dielectric layer shows a more uniform temperature distribution, but the maximum temperature is below 200 °C and the mechanical deformation is only 0.08 μm. For the plate with a composite layer, the maximum temperature exceeds 300 °C while having a smooth distribution, which verifies that the external SiO_2_ prevents the transverse heat conduction, while Si_3_N_4_ has a better heat transfer in the central region. In terms of mechanical deformation, it is noteworthy that the maximum deformation of the plate with the composite layer is reduced to 0.155 μm, which is about 82% of the MHP with a pure SiO_2_ dielectric layer. Moreover, the deformation gradient in the central region is quite less pronounced compared to the SiO_2_ layer, which suggests that the Si_3_N_4_ in the center of the composite layer mitigates the deformation caused by thermal stress.

### 3.3. The Heater Design

**Figure 5:**
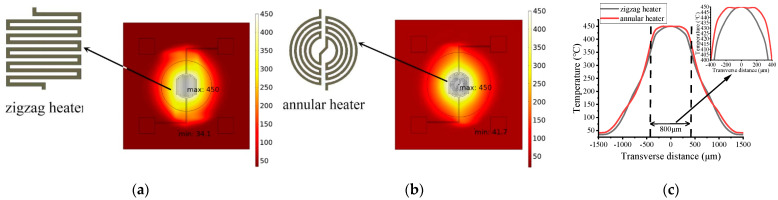
Temperature distribution of (**a**) zigzag heater, (**b**) annular heater, and (**c**) the central region with an enlarged view. The width and interval of the two heaters were set as 30 μm, the thickness was 0.2 μm, and the total length was 9400 μm. The input power was set to 41 mW.

The heat source of MHP is Joule heat generated when the DC-current flows through the Pt resistor. The designed annular heater consists of multiple concentric rings and was compared with the generally used zigzag heater shape. Figure 5a,b shows the temperature distribution of the two kinds of heater. The width and interval were set as 30 μm, thickness was 0.2 μm, and the total length was 9400 μm. The other parameters of the MHPs were set the same, and the maximum temperature of the MHP was set at 450 °C. Figure 5c more visually shows that both heaters demonstrate a similar temperature distribution, while at the central zone, the annular heater demonstrates a smoother temperature distribution.

**Figure 6:**
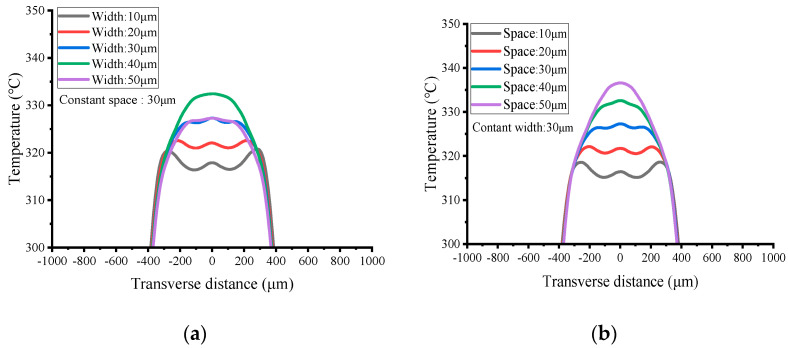
Effect of resistive (**a**) width and (**b**) space of heaters. The results are extracted from simulations of MHPs at 41 mW power input, and the other dimensions are consistent with Figure 2 except the width and space of heaters.

The effects of heater resistive track width and gap on the temperature distribution were also studied at the same power of 41 mW, and the heater radius was set as 420 μm constant. Fixed power consumption is often used in literature to study the effect of size on MHP [42,64,65,66], in order to investigate the heating efficiency. Figure 6a shows the temperature distribution at the transverse line for heater widths of 50 μm–0 μm at the constant gap, with corresponsive resistance values of 103 Ω–517 Ω. When the heater width is less than 30 μm, the uniformity of the central temperature distribution is lower. There is a good high temperature distribution when the width is set to 50 μm, and its resistance decreases to about 100 Ω. For narrower width, the uniformity is greatly descended. In addition, heaters that are too narrow are more likely to be damaged during fabrication. Figure 6b shows that a wider gap between the resistive tracks of the heater will also increase the temperature. Because the radius of the outermost layer of the annular heater is constant, increasing the gap allows the heater to cover the central area more evenly, thereby increasing the temperature. When the gap is higher or lower than 30 μm, the high temperature distribution in the center begins to become less uniform. Taken together, the MHP with a heater of width and gap of 30 μm not only achieves a uniform high temperature of 300 °C, but also allows the high temperature zone to be close to 800 μm in diameter.

### 3.4. Effect of Back-Side Etching Window Size

**Figure 7:**
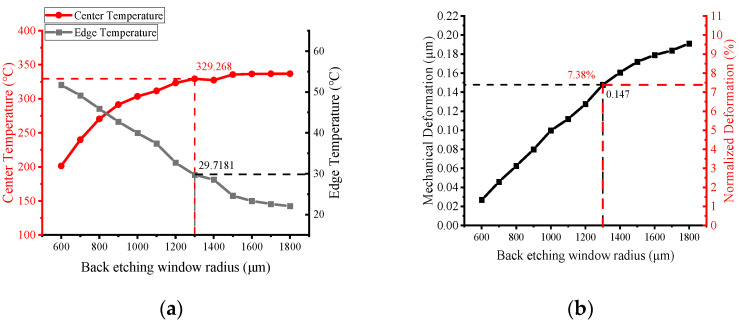
Relationship between etch window size and (**a**) temperature at the center and edge of the MHP (**b**) mechanical deformation due to thermal stress; absolute and normalized values are shown with respect to the total thickness of the MHP (2 μm). These results are extracted from simulation of the Si_3_N_4_-SiO_2_ composite film with 41 mW power input.

Back-side etching is required to reduce the heat loss of planar MHPs. The MHP model was designed as Figure 2, with both the width and gap of the heater resistance tracks setting to 30 μm; Figure 7 shows the effect of the variation of the back-side etched area on the maximum temperature and its mechanical deformation at the same power consumption. In the figures, “Center temperature” denotes the temperature at the central of the plate and “Edge temperature” denotes the temperature at the die edge. It can be concluded that the center temperature increases with the increase in the back-side etching size. The temperature tends to be flat 329 °C when the etched windows radius is larger than 1300 μm, and the edge temperature is less than 30 °C. However, the deformation increases almost linearly, as the back-side etched area increases, as shown in Figure 7b. Relatively, the optimal back-etched radius should be 1300 μm under the settings above. The mechanical deformation of the MHP is only 0.147 μm, which is 7.38% of the total thickness of the MHP.

### 3.5. Effect of Si_3_N_4_ Circular Area in the Composite Layer

**Figure 8:**
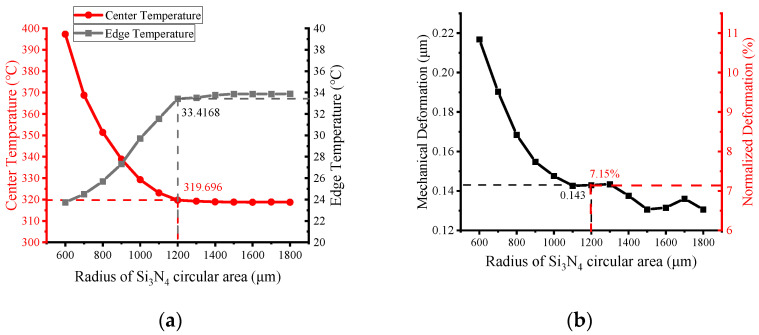
Effect of the radius of the circular Si_3_N_4_ zone on (**a**) MHP temperature distribution and (**b**) mechanical deformation due to thermal stress; absolute and normalized values are shown with respect to the total thickness of the MHP (2 μm).

**Figure 9:**
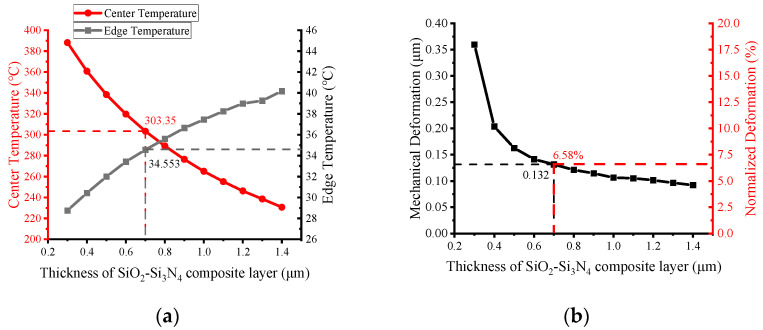
Effect of Si_3_N_4_- SiO_2_ composite layer thickness on (**a**) MHP temperature and (**b**) mechanical deformation due to thermal stress; absolute and normalized values are shown with respect to the total thickness of the MHP (2 μm).

The effect of the lateral dimensions of the Si_3_N_4_ circular region was further investigated. The relationship of the temperature, mechanical deformation, and the radius $r_{m}$ of the Si_3_N_4_ region is shown in Figure 8, with the previous optimized dimensions of the heater and the back-etching radius of the MHP. As the radius increases, the center temperature gradually decreases, while the edge temperature increases. However, after 1200 μm, the decreasing trend tends to be slow. Meanwhile, it can be observed from Figure 9b that a larger Si_3_N_4_ area can better alleviate the thermal deformation. When the Si_3_N_4_ radius is set to 1200 μm, the center temperature can reach 320 °C, while the thermal deformation is only 0.143 μm, which is 7.15% of the total thickness of the MHP.

### 3.6. Effect of the Thickness of the Dielectric Layer

Figure 9 shows the effect of the thickness of the composite layer on the temperature and mechanical deformation at the same power consumption. In this case, the total thickness of the plate was set constant of 2 μm, while the other parameters were set previous optimized values. In Figure 9a, it can be observed that increasing the proportion of the composite layer will lead to additional heat loss due to the increase in the heat conduction cross-sectional area. Therefore, as the thickness of the composite layer increases, the center temperature and the edge temperature keep decreasing and increasing, respectively. When the thickness of the composite layer exceeds 0.7 μm, the temperature of the MHP is already below 300 °C.

Figure 9b shows that increasing the thickness will alleviate the thermal deformation effect. The reason is that a thicker Si_3_N_4_ layer in the center can relieve the thermal stress more effectively. When the thickness of the composite layer exceeds 0.7 μm, the ability to relieve mechanical deformation starts to decrease. The mechanical deformation is only 0.132 μm at a center temperature of 303 °C, which is 6.58% of the total thickness of the MHP.

### 3.7. Power Optimization Analysis

**Figure 10:**
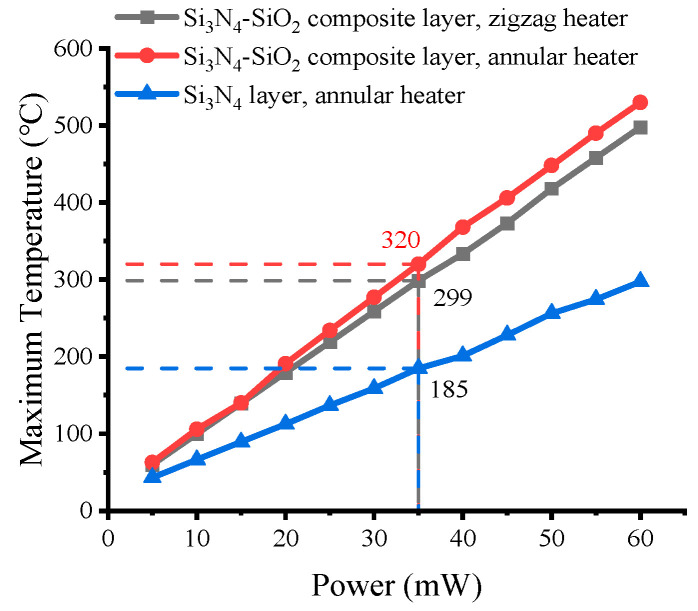
Effect of maximum temperature on power input of MHP with Si_3_N_4_-SiO_2_ composite layer and zigzag heater, MHP with Si_3_N_4_-SiO_2_ composite layer and annular heater, and MHP with Si_3_N_4_ layer and annular heater.

Figure 10 shows the power consumption of three types of MHPs without the signal electrodes. All MHPs use optimized dimensions for all layers. The MHP with a Si_3_N_4_-SiO_2_ composite layer shows clearly higher temperature than the MHP with a Si_3_N_4_ layer of the same dimensions. The designed membrane with an annular heater can reach 320 °C at 35 mW of power. The MHP with a zigzag heater can reach 299 °C, which is a bit lower than the annular heater at the same power.

Finally, the parameters of the designed MHP are extracted and listed in the last row of Table 1. With the heating area of 0.5539$\;{\mathrm{mm}}^{2}$, the MHP designed with the annular heater and composite layer in this paper requires only 35.2 mW heating power to reach 300 °C. The specific area power, 0.0064 (mW/1000 μm^2^), which is the ratio of power per heated area, is greatly lower than other MHPs in the literature, as listed in Table 1. Hence, it can be observed that the designed MHP does demonstrate a higher power conversion efficiency. This further indicates that the proposed design of Si_3_N_4_-SiO_2_ transversal composite dielectric layer effectively reduces the heat loss.

## 4. Conclusions

The reduction of the heat loss of MHPs is an active research topic. To reduce the heat loss of planar type MHPs, a Si_3_N_4_-SiO_2_ composite layer is proposed to form a new design of MHP. Detailed analyses and systematic simulations verify that the designed MHP can effectively block the transverse thermal conduction loss and obtain a more uniform high temperature region, greatly mitigating the mechanical deformation due to thermal stress. In addition, an optimized planar MHP is obtained. It can heat an area of about 0.5$\;{\mathrm{mm}}^{2}$ to 300 °C with a power consumption as low as 35.2 mW and mechanical deformation as low as 0.132 μm, about 6.58% relative deformation to the plate. The specific power to the heated area is only 0.0064 (mW/1000 μm^2^), which is much lower than the values in literature listed in Table 1, demonstrating that a very high heating efficiency is obtained by this design.

## Figures and Tables

**Table 1:** Comparison of various MHP designs that have been reported in literature. If exact values are not given, they have been deduced from the information given in the specific literature. (Note: For suspended devices, the total device area is often not given and is therefore not included for some of the devices).

Year	Active Area $\left(1000\;\mu m^{2}\right)$	MHP Area $\left(1000\;\mu m^{2}\right)$	Power at 300 °C (mW)	Power/ Heater Area ^1^	Already Fabricated?	Planar/ Suspended	Material of Heater	Material of Plate	Ref.
2002	10		30	3	Yes	Suspended	Poly Si	SiO_2_	[26]
2010	140.6		13.5	0.096	Yes	Suspended	3C-SiC	AlN/SiC	[27]
2012	108	1000	22.68	0.21	Yes	Planar	Molybdenum	SiO_2_/SiN	[28]
2014	10		19	1.9	Yes	Suspended	W	SiO_2_	[29]
2014	10	267.3	27.66 (367 °C)	2.77	No	Planar	Pt	SiO_2_/Si_3_N_4_	[30]
2016	846.4		100	0.118	Yes	Planar	ITO	Si_3_N_4_	[19]
2016	17.6	250	14	0.79	Yes	Planar	W	SiO_2_	[20]
2018	250		4	0.016	No	Suspended	Pt	Si_3_N_4_/SiC	[21]
2018	6.4	22.5	10	1.56	Yes	Suspended	Pt	SiO_2_	[22]
2018	10	90	13	1.3	Yes	Suspended	W	SiO_2_/Si_3_N_4_	[23]
2018	101.7	540	8	0.079	No	Suspended	Pt	SiO_2_/Si_3_N_4_	[31]
2019	0.032		7	218.75	Yes	Suspended	Pt	Si	[18]
2019	250	490	30	0.12	Yes	Planar	Pt	SiN/SiO_2_	[32]
Thiswork	553.9	6154.4	35.2	0.064	No	Planar	Pt	SiO_2_-Si_3_N_4_	

^1^ The unit of “Power/Heater area” is mW/1000 μm^2^.

**Table 2:** Comparison of MHPs at 35 mW heating power.

Type	Structure	Heat Area (μm)	Center Temperature (°C)	Maximum Deformation (μm)
A	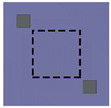	800 × 800	158	0.052
B	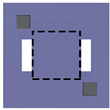	800 × 800	174	0.223
C	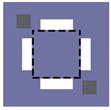	800 × 800	199	0.228
D	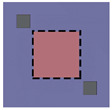	800 × 800	275	0.098

**Table 3:** Material properties used in the simulations [26,42,60,61,62,63].

Material	Pt	Si	SiO_2_	Si_3_N_4_	Au
Thermal conductivity coefficient (W m^−1^ K^−1^)	71.6	130	1.4	20	317
Range of thermal conductivity coefficient found in literature (W m^−1^ K^−1^)	70–72	130	1.2–1.4	9–30	317
Electrical conductivity (S m^−1^)	8.9 × 10^6^	1 × 10^−12^	1 × 10^−10^	1 × 10^−2^	45.6 × 10^6^
Heat capacity (J Kg^−1^ K^−1^)	133	703	730	700	129
Density (Kg m^−3^)	24,150	2329	2200	3100	19,300
Thermal expansion coefficient (10^−6^ K^−1^)	8.80	2.6	0.5	2.3	14.2

## Data Availability

The data presented in this paper are available from the corresponding author upon request.
